# Modulation of the oxidative damage, inflammation, and apoptosis-related genes by dicinnamoyl-L-tartaric acid in liver cancer

**DOI:** 10.1007/s00210-023-02511-8

**Published:** 2023-05-09

**Authors:** Alaa Elmetwalli, Shimaa Mustafa Hashish, Mervat G. Hassan, Mohammed Abu El-Magd, Sabry Ali El-Naggar, Amina M. Tolba, Afrah Fatthi Salama

**Affiliations:** 1 Department of Clinical Trial Research Unit and Drug Discovery, Egyptian Liver Research Institute and Hospital (ELRIAH), Mansoura, Egypt; 2 Division of Biochemistry, Department of Chemistry, Faculty of Science, Tanta University, Tanta, Egypt; 3 Department of Botany and Microbiology, Faculty of Science, Benha University, Benha, 33516 Egypt; 4 Department of Anatomy, Faculty of Veterinary Medicine, Kafrelsheikh University, Kafrelsheikh, Egypt; 5 Zoology Department, Faculty of Science, Tanta University, Tanta, Egypt; 6 Department of Anatomy, Faculty of Medicine for Girls, Al-Azhar University, Cairo, Egypt

**Keywords:** Apoptosis, Liver cancer, Dicinnamoyl-L-tartaric acid, Sorafenib, Oxidative stress

## Abstract

Cancer cells can become resistant to existing treatments over time, so it is important to develop new treatments that target different pathways to stay ahead of this resistance. Many cancer treatments have severe side effects that can be debilitating and even life-threatening. Developing drugs that can effectively treat cancer while minimizing the risks of these side effects is essential for improving the quality of life of cancer patients. The study was designed to explore whether the combination of dicinnamoyl-L-tartaric (CLT) and sorafenib ((SOR), an anti-cancer drug)) could be used to treat hepatocellular carcinoma (HCC) in the animal model and to assess whether this combination would lead to changes in certain biomarkers associated with the tumour. In this study, 120 male mice were divided into 8 groups of 15 mice each. A number of biochemical parameters were measured, including liver functions, oxidative stress (malondialdehyde, (MDA); nitric oxide (NO)), and antioxidative activity (superoxide dismutase (SOD), and glutathione peroxidase (GPx)). Furthermore, the hepatic expressions of *Bax*, *Beclin1*, *TNF*-*α*, *IL1β*, and *BCl-2* genes were evaluated by qRT-PCR. The combination of SOR and CLT was found to reduce the levels of liver enzymes, such as AST, ALT, ALP, and GGT, and reduce the pathological changes caused by DAB and PB. The upregulation of *TNF-α*, *IL1β*, and *Bcl-2* genes suggests that the CLT was able to initiate an inflammatory response to combat the tumor, while the downregulation of the *Bax* and *Beclin1* genes indicates that the CLT was able to reduce the risk of apoptosis in the liver. Furthermore, the combination therapy led to increased expression of cytokines, resulting in an enhanced anti-tumor effect.

## Introduction

In the world, hepatocellular carcinoma (HCC), a kind of liver cancer, is the second most prevalent cause of cancer-related mortality (Llovet et al. 2022). For more than 90% of HCC cases, chemotherapy and immunotherapy are currently the best options for their treatment. Since sorafenib (SOR) was the first multi-tyrosine kinase inhibitor to be approved for the treatment of HCC, it has been the gold standard of care for patients with advanced HCC for almost ten years (Pang et al. 2022). Inflammation has been linked to tumor promotion, progression, and metastasis, as well as resistance to chemotherapy and radiotherapy. It has been shown to increase the risk of developing certain types of cancer, as well as influence the prognosis of cancer patients (Chen and Song 2022). The *IL-6* antibody helps to reduce the levels of *IL-6* in the body, which in turn helps to reduce the growth and spread of tumor cells. This in turn can lead to improved survival outcomes and enhanced anti-tumor effects when combined with chemotherapy, natural therapy, or SOR (Pradhan et al. 2023). Furthermore, targeting transforming growth factor (TGF)-β, either through inhibition or suppression, could potentially be an effective way to reduce cancer progression and increase the effectiveness of existing anti-tumor therapies such as epidermal growth factor receptor (EGFR)-targeted therapy (Pang et al. 2022). Although SOR significantly extends advanced HCC patients’ median survival rates (Zeng et al. 2021), its benefits are limited. Furthermore, SOR resistance remains an obstacle to efficient chemotherapy for HCC (Tang et al. 2020). Moreover, the long-term side effects of SOR can cause serious health problems, such as fatigue, anorexia, weight loss, and hand-foot syndrome (HFS), with HFS being the most common side effect (Kobayashi et al. 2019). This means that despite SOR’s efficacy in extending survival times, it also carries significant risks which should be weighed against its potential benefits (Zou et al. 2021).

The adverse effects associated with SOR are typically transient and can usually be managed, but some studies suggest that natural supplements are thought to reduce the severity of side effects associated with SOR therapy by providing additional antioxidant and anti-inflammatory agents that can help to mitigate the potential adverse effects of this medication (Man et al. 2021; Attia et al. 2022; El-Shehawy et al. 2023; Nasser et al. 2021). Additionally, they can help to enhance the efficacy of the drug by providing additional nutrients that can aid in the metabolism and absorption of the medication (Yao et al. 2018). Combination therapies, such as traditional herbal medicines or natural compounds, have emerged as novel therapeutic approaches for reducing adverse events and improving clinical outcomes in SOR therapy (Fornari et al. 2021; Elmalla et al. 2021).

Hydroxycinnamic acid (HCA) is one of the phytochemicals that are particularly interested in employing as part of an innovative approach for the prevention and treatment of HCC. HCA is one of the most prevalent and extensively distributed classes of phenolic acids in plants (Hoa et al. 2022). Scientists have been interested in cinnamon acid (a derivative from HCA) because of its antioxidant, antiproliferative, antiangiogenic, and antitumor properties (Sadeghi et al. 2019). Thus, a wide variety of derivatives of dicinnamoyl-L-tartaric (CLT) have been investigated for their anti-tumor properties (Deng et al. 2022). Several studies have shown that HCAs, including caffeic, ferulic, and coumaric acids, can be effective in fighting cancer, including colon cancer (Rosa et al. 2016), adenocarcinoma (Murad et al. 2015), and hepatocarcinoma (Espíndola et al. 2019). Moreover, both in vitro and in vivo colon cancer cells treated with HCA were activated by outspread protein response-mediated apoptosis, which is usually unregulated in this disease (Fantini et al. 2015). Furthermore, HCA reduced inflammation by lowering levels of *IL-6*, *COX-II*, and *TNF-β* expression (Wang et al. 2015; Park et al. 2017).

Therefore, to determine the full effects of such combination therapy, further studies are required on CLT as a novel derivative of HCA and SOR. In light of this, we hypothesized that CLT and/or SOR could also inhibit hepatocellular carcinoma growth by upregulating tumour suppressor proteins and suppressing oncogenic proteins. For validating this hypothesis, we conducted a detailed in vivo study to investigate whether CLT reduces SOR resistance and mitigates liver inflammation induced by dimethylaminoazobenzene (DAB).

## Materials and methods

### Chemicals

CLT was purchased from Sigma Aldrich (St. Louis, MO, USA). The commercial kits of ALT, AST, and GLT were obtained from Biomed Company (Cairo, Egypt). The albumin kit was purchased from Biomed Diagnostics Company, Egypt. The commercial kits of malondialdehyde (MDA), superoxide dismutase (SOD), and glutathione peroxidase (GPx) were purchased from Life Science Inc. RNA extraction and Thermo Scientific, Fermentas, supplied purification, cDNA synthesis kits, and SYBR Green.

### Animals and ethics statement

This approval was granted based on the guidelines for the care and use of animals set forth by Tanta University’s Faculty of Science (IACUC). The guidelines include guidelines regarding animal care, housing, and procedures that minimize the suffering and distress of animals. IACUC has approved this protocol with the approval number (IACUC-SCI-TU-0133). There was strict adherence to the ARRIVE guidelines throughout all of the procedures carried out during the research to ensure that the animals were receiving the best possible care during the process. Moreover, all procedures are conducted ethically and humanely at all times. As a part of the experiment, 120 male Swiss albino mice of the weight range of 20–25 g and aged between 10 and 12 weeks were used. A temperature-controlled environment was provided to the mice during the experiment, with a 24-h cycle. All animals received a normal diet and free access to water ad libitum.

### The hepatocellular carcinoma animal model

According to the method described by Biswas et al. (2008), the development of the hepatocellular carcinoma animal model was induced following the administration of DAB (15 mg/kg body weight) five times a week as the initiator and PB as the promoter at a dose of 0.05% (five times a week) as the promoter for 2 months. This was followed by careful monitoring of the animals for the detection of any signs of tumor formation.

### Experimental design

**Fig. 1:**
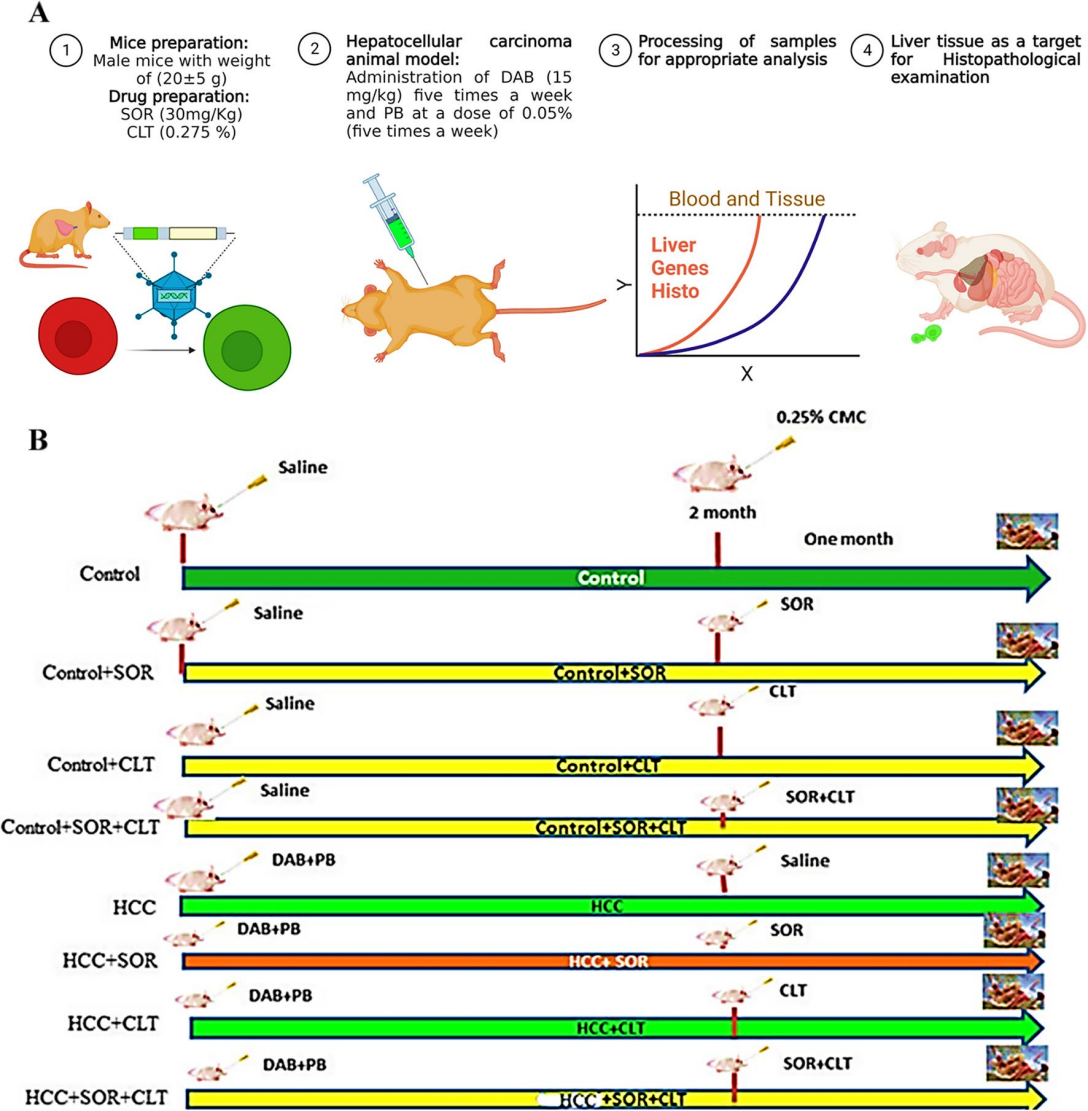
**A** Flowchart outlines all the steps taken to complete the process, as well as the inputs and outputs of each step. It provides a visual representation of the steps taken and is useful for tracking progress and ensuring that all steps are completed correctly. **B** Animal grouping design: each group contained 15 mice; Group I (negative control group); Group II (control + SOR); Group III (control + CLT); Group IV (control + SOR + CLT); Group V (HCC group); Group VI (HCC + SOR); Group VII (HCC + CLT); Group VIII (HCC + SOR + CLT). Abbreviation: CMC: carboxymethylcellulose; SOR: sorafenib; CLT: dicinnamoyl-L- tartaric acid; HCC: hepatocellular carcinoma

Each group of mice contained 15 mice, and they were divided after a week of acclimatization as follows: *Group I* (negative control group): Oral saline was administered to mice through a stomach tube, and 0.25% carboxymethyl cellulose (CMC) was administered as a vehicle (5 times a week for 2 months). *Group II* (control + SOR): Normal saline was administered to mice for 2 months, and then, SOR was administered orally by a stomach tube five times per week for one month at a dose of 30 mg/kg (Attia et al.^2022^). *Group III* (control + CLT): CLT was administered orally (0.0275%) as previously described by Zych et al. (2019) with minor modification via a stomach tube five times a week for 1 month to mice that had received normal saline for two months. *Group IV* (control + SOR + CLT): For 2 months, mice were given normal saline, and then, for 1 month, they were given (SOR + CLT) orally through a stomach tube. *Group V* (HCC group): Mice were treated with DAB and PB as previously described by Biswas et al. (2008). *Group VI* (HCC + SOR): SOR was administered orally to HCC animal models for 1 month at the same dose previously mentioned. Group VII (HCC + CLT): HCC animal models received CLT orally by stomach tube at the same previously mentioned dose for one month. *Group VIII* (HCC + SOR + CLT): The experimental HCC mice received a combination of SOR and CLT given by stomach tube at the same formerly stated dose for one month. An overview of the experiment design and workflow diagram can be seen in Fig. 1A–B.

### Blood and tissue samples

Upon completion of the experiment, mice fasted for 12 h and euthanasia was applied by isoflurane anesthesia. After collecting blood from the mice from the tail vein of the mice, the samples were centrifuged for 10 min at 2000 g for 20 min. Upon separation and collection of sera, Eppendorf tubes were placed in − 20 °C freezers for biochemical analysis. Also, three portions of the liver were separated and weighed. RNA was extracted from the 1st and 2nd portions of hepatic tissue and stored at − 80 °C before RNA extraction, real-time PCR, and assessment of lipid peroxidation, antioxidant status, and lipid peroxidation levels. The third portion was preserved in 10% formalin for histological analysis.

### Serum biochemical assessment

Aspartate aminotransferase (AST), serum alanine aminotransferase (ALT) (Bergmeyer et al. 1976), γ-glutamyl transferase (GGT) (Szasz 1974), albumin (Doumas et al. 1971), and alkaline phosphatase (ALP) were assessed by quantitative colorimetric methods (Patlolla et al. 2015). By using the ELISA sandwich technique, the alpha-fetoprotein (AFP) was determined following (Belfield and Goldberg 1971). All the procedures were done following the manufacturer’s instructions.

### The hepatic oxidative/antioxidant assessment

The liver homogenate was made in the following manner; liver sections were homogenized in 5 ml potassium phosphate buffer (100 mM, pH 7), centrifuged at 1520 g for 15 min, and then collected the supernatant in a clean, dry Eppendorf tube and kept at 80 °C until further assays. The hepatic nitric oxide (NO) (Montogomery and Dymock 1961), malondialdehyde (MDA) (Mesbah et al. 2004), superoxide dismutase (SOD), and glutathione peroxidase (Gpx) activity were assessed following the methods of (Nishikimi et al. 1972) (Paglia and Valentine 1967), respectively.

### Histopathology study

The liver tissue from every mouse in the experiment was prepared and sectioned for histological analysis. An ethyl alcohol solution with varying concentrations of neutral formalin was used to fix and dry the hepatic tissue. A xylene wash was performed, and then, the tissue was embedded in paraffin blocks. All sections are made by 5 µ and stained with hematoxylin and eosin (H&E) as previously described by Suvarna et al. (2013). All experimental groups were photographed with a light microscope for their histopathological alterations.

### qRT-PCR

The Nanodrop 2000c measures the concentration and purity of the RNA, ensuring that the 5 µg of RNA used in the next step has the necessary quality for the experiment. The reverse transcriptase kits then convert the RNA into complementary DNA, which can then be used for further analysis (Verma 1981). The mixture was incubated at 42 °C/60 min and was stopped by heating it for 10 min at 70 °C. By using *β-actin*, as an internal reference gene, we evaluated the mRNA expressions of target genes in the liver tissue using Step OnePlus real-time PCR system (Applied Biosystems, USA) with SYBR Green and gene-specific primers. The *TNF-α*, *Il-1*, *TGF-β*, *Bax*, *Bcl2*, and *Bec1* genes’ relative expression was evaluated using cDNA as a template. Table 1 provides a list of the primer sequences. The reaction cycle was 95 °C for 10 min, followed by 40 cycles at 95 °C for 15 s, and 60 °C for 30 s. The Ct value analysis calculates the fold change in gene expression (Livak and Schmittgen 2001).

**Table 1:** The real-time PCR primer sequences

	Sense	Antisense
TNF-α	TTCTGTCTACTGAACTTCGGGGTGA TCGGT CC	GTATGAGATAGCAAATCGGCTGACG GTGTGGG
Il-1β	AAGGAGAACCAAGCAACGACAAAA	TGGGGAACTCTGCAGACTCAAACT
TGF- B	TGGAGCAACATGTGGAACTC	GTCAGCAGCCGGTTACCA
Bax	CTACAGGGTTTCATCCAG	CCAGTTCATCTCCAATTCG
Bcl2	GTGGATGACTGAGTACCT	CCAGGAGAAATCAAACAGAG
Bec1	AATCTAAGGAGTTGCCGTTATAC	CCAGTGTCTTCAATCTTGCC
*β-actin*	CCTGTATGCCTCTGGTCGTA	CCATCTCCTGCTCGAAGTCT

### Statistical analysis

The data were examined using the GraphPad Prism 8.0 software. The experimental data were expressed using mean ± SEM. The data were evaluated using ANOVA following the Tukey test for multiple comparisons. Statistical significance was defined as *p* < 0.05 (Armitage et al. 2008).

## Results

### Impact of CLT and/or SOR on biochemical parameters

The administration of DAB/PB triggered an increase in the activity of enzymes such as AST, ALT, GGT, and ALP, which are usually elevated in response to damage or stress on the liver. This increase in enzyme activity is associated with a decrease in albumin concentration, which is indicative of liver damage. When SOR and/or CLT were administered after HCC induction, there was a significant reduction in AST, ALT, GGT, and ALP activity. In addition, there was a significant increase in albumin levels in comparison with the HCC group. SOR and CLT are natural compounds that have been found to have hepatoprotective effects, which means that they can protect the liver from damage. This is evidenced by the decrease in AST, ALT, GGT, and ALP activity, as well as the increase in albumin concentration, which are all indicators of liver health. The combination of the two treatments resulted in a greater reduction in symptoms than either treatment alone. This indicates that the two treatments work synergistically, and when used together, they are more effective than when used separately as illustrated in Table 2.

**Table 2:** The modulatory impact of CLT and/or SOR on hepatic biomarker function

Groups	AST (U/L)	ALT (U/L)	GGT (U/L)	Albumin (g/dl)	ALP (U/L)
Control	35.65 ± 2.11d	27.47 ± 1.42d	9.40 ± 0.49d	3.15 ± 0.14a	222.36 ± 10.42e
SOR	41.49 ± 2.40d	33.62 ± 1.75d	9.27 ± 0.53d	2.75 ± 0.12ab	254.60 ± 11.05cde
CLT	33.02 ± 1.91d	26.29 ± 1.29d	9.90 ± 0.58d	3.07 ± 0.13a	215.42 ± 9.87e
SOR + CLT	37.36 ± 2.20d	30.17 ± 1.84d	9.61 ± 0.42d	2.92 ± 0.13ab	243.50 ± 10.36de
HCC	128.47 ± 7.48a	117.15 ± 5.80a	39.26 ± 1.52a	1.10 ± 0.05e	398.36 ± 15.13a
HCC + SOR	90.50 ± 4.62b	84.36 ± 2.82b	24.06 ± 1.24c	1.73 ± 0.07d	337.28 ± 12.39b
HCC + CLT	84.05 ± 3.69bc	73.90 ± 2.45b	28.57 ± 1.37b	2.15 ± 0.09 cd	302.57 ± 7.52bc
HCC + SOR + CLT	67.45 ± 3.26c	56.61 ± 2.35c	20.15 ± 1.01c	2.58 ± 0.08bc	287.83 ± 6.86 cd

The data were displayed as mean ± SEM. Small (a–e) letters depicted the statistically significant change at *P* ≤ 0.05 using ANOVA followed by post hoc Tukey test for multiple comparisons test. The significant changes are expressed by different letters in the same columns. Abbreviation: SOR, sorafenib; CLT, dicinnamoyl-L-tartaric acid; HCC, hepatocellular carcinoma; ALT, alanine aminotransferase; AST, aspartate aminotransferase; GGT, gamma-glutamyl transferase; ALP, alkaline phosphatase

### Impact of CLT and/or SOR on Serum AFP

**Fig. 2:**
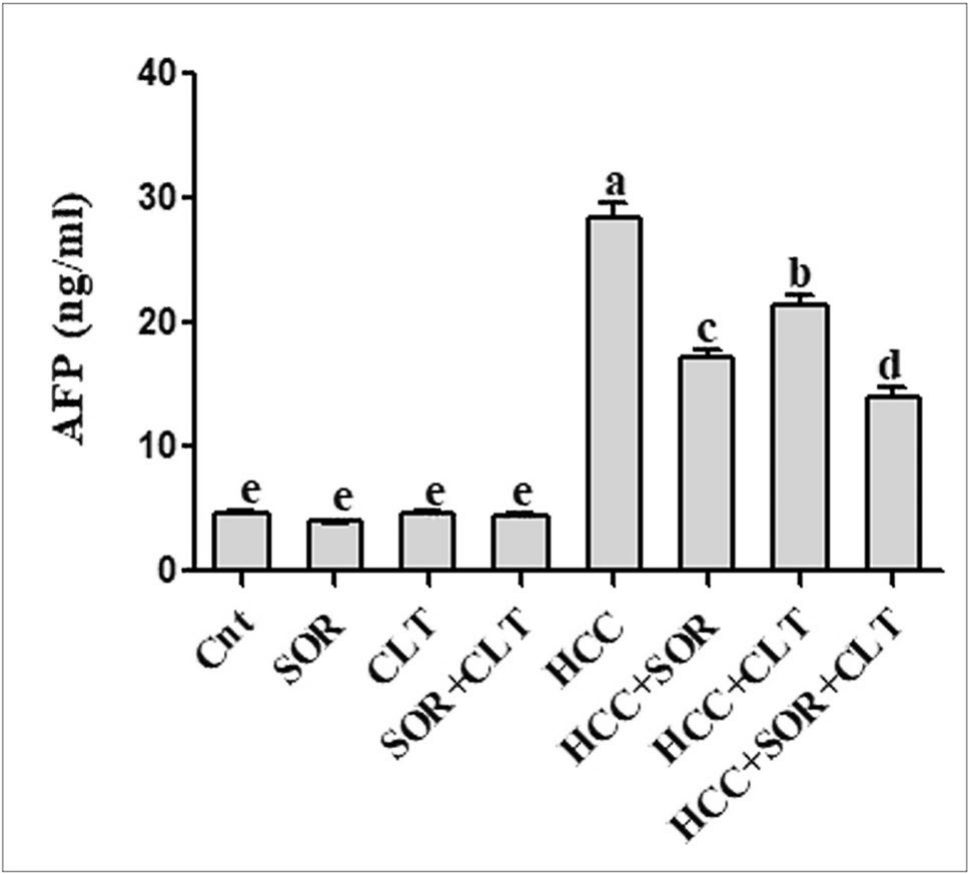
The serum α-fetoprotein (AFP) level in different studied groups. Data were expressed as mean ± SEM (*n* = 15). Small (a–e) letters depicted the statistically significant change at *P* ≤ 0.05 using ANOVA followed by post hoc Tukey test for multiple comparisons test. The significant changes are expressed by different letters above the columns. Abbreviation: SOR: sorafenib; CLT: dicinnamoyl-L- tartaric acid; HCC: hepatocellular carcinoma

The data indicated that there is no significant change in serum AFP levels between the untreated control group and the treated control groups, while the serum AFP was significantly elevated in the DAB/PB-induced HCC group compared to the untreated group. SOR and CLT, either alone or combined, are effective in suppressing elevated serum AFP levels, indicating a decrease in inflammation induced by HCC. Moreover, the combination of the two treatments is more effective in suppressing inflammation than either treatment alone as shown in (Fig. 2).

### Impact of CLT and/or SOR on hepatic oxidative and antioxidant status

**Fig. 3:**
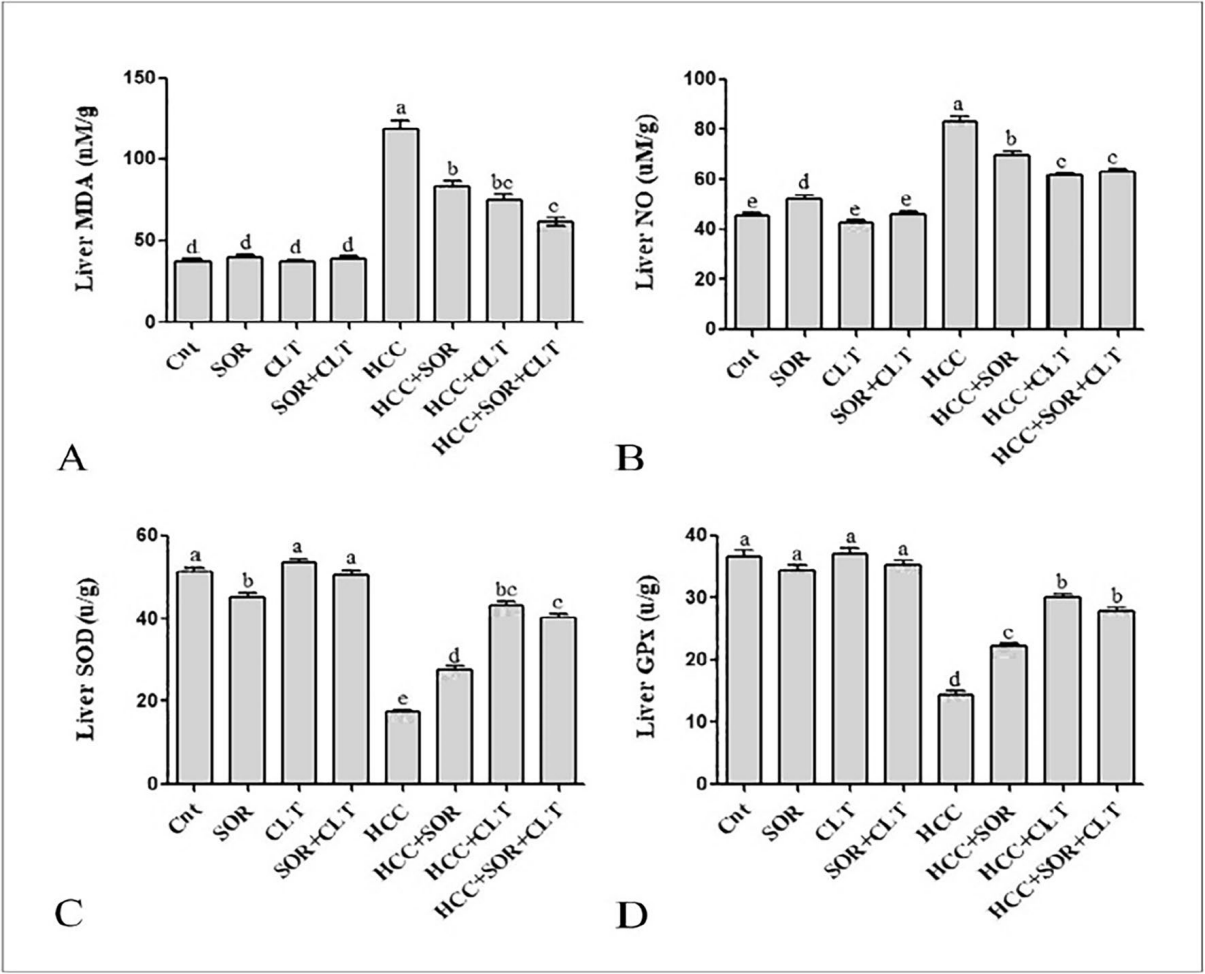
**A** Liver homogenate for assessment of malondialdehyde (MDA) level. **B** Nitric oxide (NO) concentration. **C** Superoxide dismutase (SOD). **D** Glutathione peroxidase (GPX). Data were expressed as mean ± SEM (*n* = 15). Small (a–e) letters depicted the statistically significant change at *P* ≤ 0.05 using ANOVA followed by post hoc Tukey test for multiple comparisons test. The significant changes are expressed by different letters above the columns. Abbreviation: SOR: sorafenib; CLT: dicinnamoyl-L- tartaric acid; HCC: hepatocellular carcinoma

DAB/PB injection significantly increased the level of MDA in the hepatic tissues while decreasing SOD and GPx activity compared to positive control groups. This indicates that the injection of DAB/PB has a pro-oxidant effect on the liver, which could be due to an increase in the production of reactive oxygen species, leading to an increase in the levels of MDA and NO and a decrease in the activity of antioxidant enzymes such as SOD and GPx. Furthermore, our results indicate that SOR and/or CLT treatment may be beneficial in reducing oxidative damage in HCC cells. The decrease in MDA and NO levels, as well as the increase in the activity of SOD and GPx, suggests that SOR and/or CLT may be effective in decreasing oxidative stress in HCC cells. The combination of SOR and CLT allows for a more comprehensive treatment that provides more targeted symptom relief. It also encourages better adherence to the treatment plan, leading to better overall outcomes as depicted in (Fig. 3).

### Histopathological analysis

**Fig. 4:**
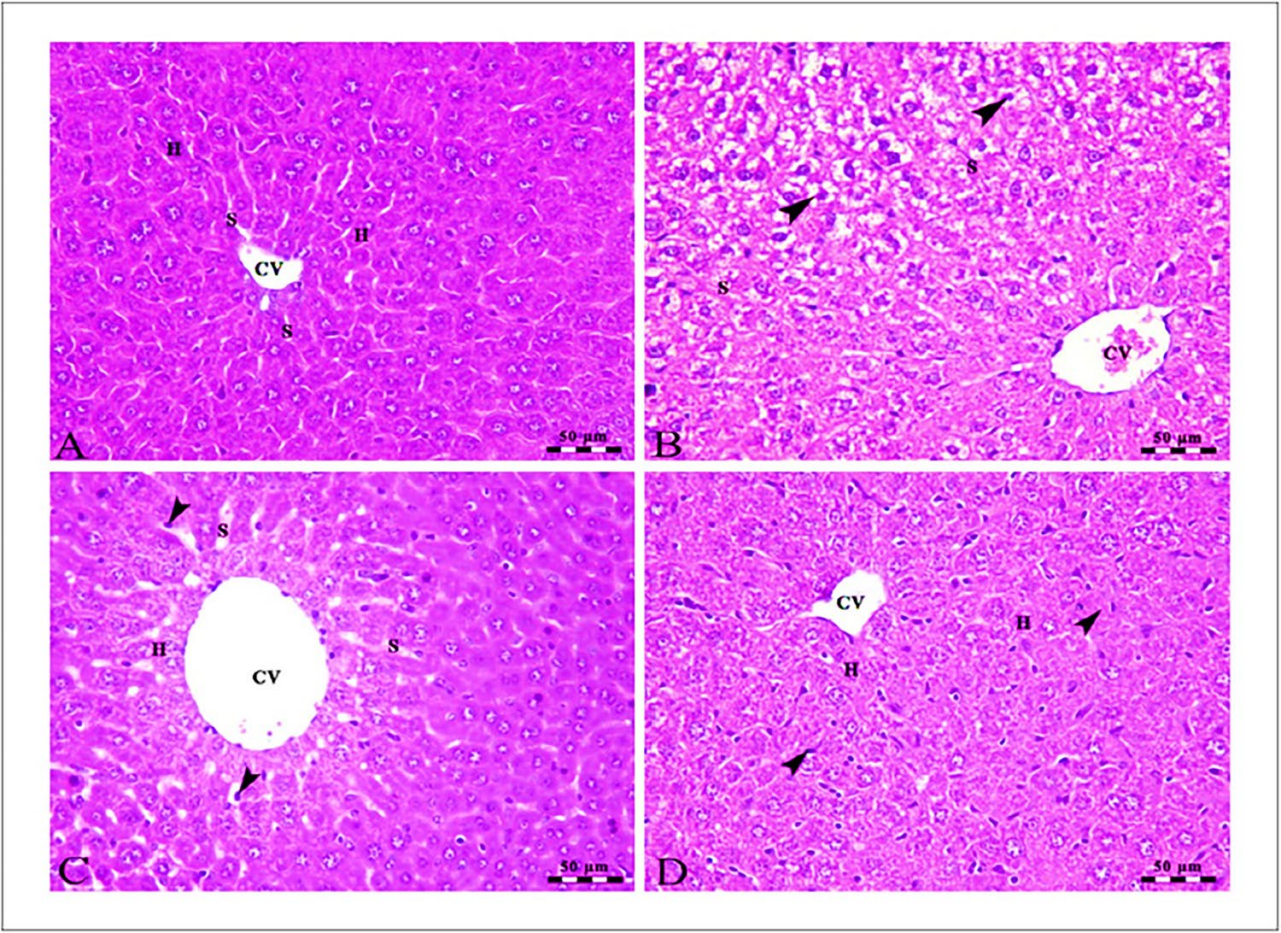
**A** Photomicrographs of the liver of the control group showing intact polyhedral-shaped hepatocytes (H) with a rounded centrally located nucleus arranged in a cord-like pattern and separated by narrow blood sinusoids (S) which radiates from the intact central vein (CV). **B** The centro lobular area of the liver of the SOR-treated group showing mild swelling and vacuolar degeneration of some hepatocytes at the periphery of the lobule (arrowheads) besides congestion in both central veins (CV) and hepatic sinusoids (S). **C** The liver of the CLT-treated group showing mild micro steatosis in the hepatocytes (H), mild dilated central vein (CV), and blood sinusoids (S) in addition to the presence of Kupffer cells (arrowheads). **D** The centro lobular area of the liver of the SOR + CLT-treated group showing central vein (CV), intact hepatocytes arranged in cords (H), and the presence of Kupffer cells activity (arrowheads). Stain H&E. Scale bars = 50 µm

In Fig. 4, the liver cells of the control group were healthy and functioning properly, as they were arranged in the typical clustered pattern and there was clear evidence of blood flow through the sinusoids which radiated from the central vein (Fig. 4A). SOR treatment can cause mild damage to the hepatocytes, which causes swelling and vacuolar degeneration. This damage can be seen in the centro lobular area, as evidenced by the arrowheads, and is also indicated by the congestion in the central veins and hepatic sinusoids (Fig. 4B). The mild micro-steatosis indicates that there is an accumulation of fat droplets in the hepatocytes, which is likely due to the CLT treatment. The dilated central vein suggests that there is a disruption in the normal flow of blood in the liver, and the presence of Kupffer cells indicates that the liver is responding to inflammation or damage (Fig. 4C). The presence of Kupffer cells indicates that the liver is actively working to remove toxins and other harmful substances from the body. Intact hepatocytes arranged in cords show that the liver is functioning properly and not damaged by the SOR + CLT-treated group (Fig. 4D).

**Fig. 5:**
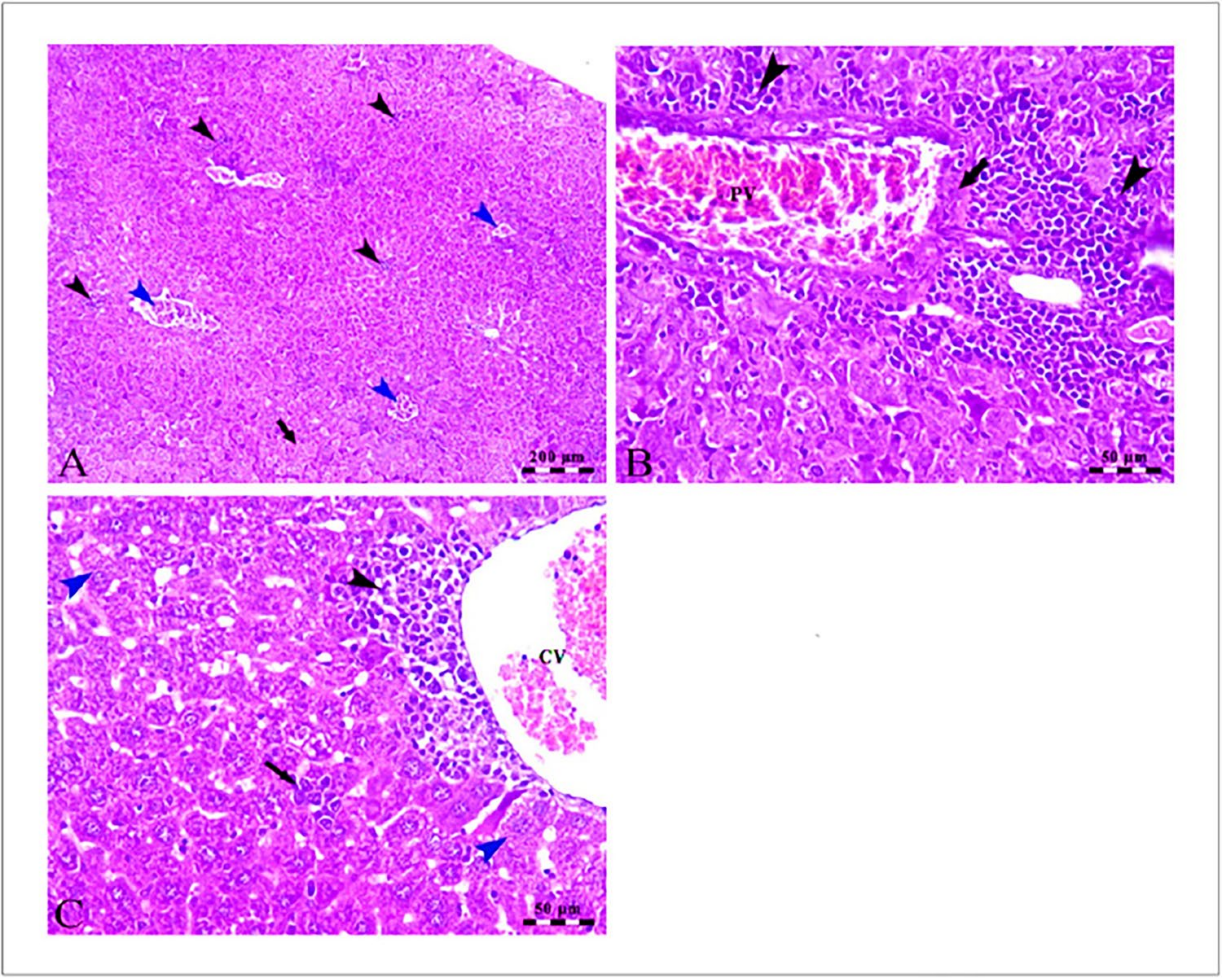
**A** Photomicrographs of the liver of HCC-induced group showing loss of hepatic architecture with the presence of numerous aggregations of lymphoid stroma (black arrowheads), congestion of hepatic blood vessels (blue arrowheads) atypia, and degeneration of some hepatocytes (arrow). **B** The portal area of the liver of the HCC-induced group showing congestion of the portal vein (PV) besides increased thickness of its wall (arrow) in addition to the presence of large periportal lymphoid element aggregation (arrowheads). **C** The centro lobular area of the liver of HCC induced group showing dilation and congestion of the central vein (CV), perivascular aggregation of lymphoid elements (black arrowhead), atypia of hepatocytes (black arrow), and ballooning and microsteatosis of some hepatocytes (blue arrowheads). Stain H&E. Scale bars = 50 µm

**Fig. 6:**
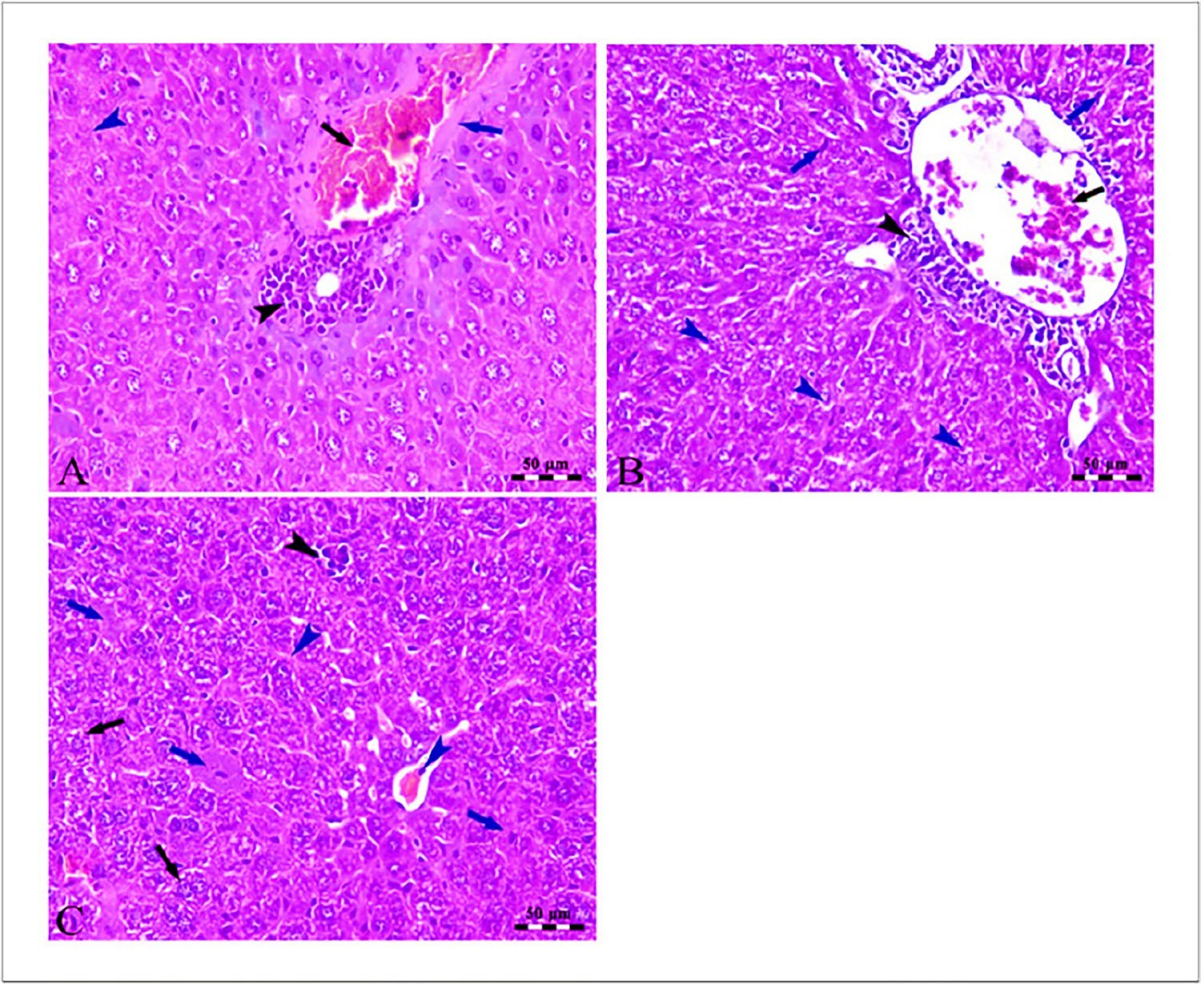
**A** Photomicrographs of the periportal area of the liver of the HCC-induced SOR treated group showing severe congestion of the portal vein (black arrow), thickening of the wall of the portal vein (blue arrow), small aggregation of lymphoid elements (black arrowhead), and nuclear pyknosis and hepatocellular degenerations (blue arrowheads). **B** The liver of the HCC induced + dicinnamoyl-L-tartaric acid treated group showing moderate congestion of hepatic blood vessels (black arrow) with the presence of small perivascular lymphoid elements (black arrowhead) beside microsteatosis of hepatocytes (blue arrowhead) and Kupffer cells activity (blue arrows). **C** The liver of the HCC induced + dicinnamoyl-L-tartaric acid + sorafenib treated group showing congestion of hepatic blood vessels and sinusoids (blue arrowhead), ballooning and microsteatosis of some hepatocytes (black arrows), and small areas of hepatocellular degeneration (blue arrows) in addition to the presence of small lymphoid elements aggregations (black arrowhead). Stain H&E. Scale bars = 50 µm

In Fig. 5A, the photomicrographs show changes in the liver of the HCC-induced group compared to the control group. The changes include an increase in the lymphoid stroma, congestion of hepatic blood vessels, and atypia and degeneration of some hepatocytes. These changes suggest that HCC may be responsible for the observed changes in the liver. Further, HCC disrupted the normal functioning of the liver, resulting in congestion of the portal vein as well as an increase in the size and number of the lymphoid elements surrounding the vein (Fig. 5B). In addition, The dilation and congestion of the central vein suggest that blood is not able to flow properly through the area, likely due to the presence of the atypical hepatocytes and the accumulation of lymphoid elements. The ballooning and microsteatosis of some of the hepatocytes are signs of cell damage due to the presence of HCC (Fig. 5C). In Fig. 6A, the photomicrograph provides evidence that treatment of SOR can lead to severe congestion of the portal vein, as well as wall thickening, lymphoid element aggregation, nuclear pyknosis, and hepatocellular degeneration. Figure 6 B provides evidence that treatment with HCC + CLT can reduce the severity of congestion of hepatic blood vessels and also reduce the microsteatosis of hepatocytes, as well as increase the activity of Kupffer cells. HCC mice treated with combination treatment had improved cellular histology with minimal hepatic alterations, including coagulative and vacuolar degeneration in hepatocytes, and a higher improvement in hepatic histology was found as presented in Fig. 6C.

### Gene expressions by qRT-PCR

**Fig. 7:**
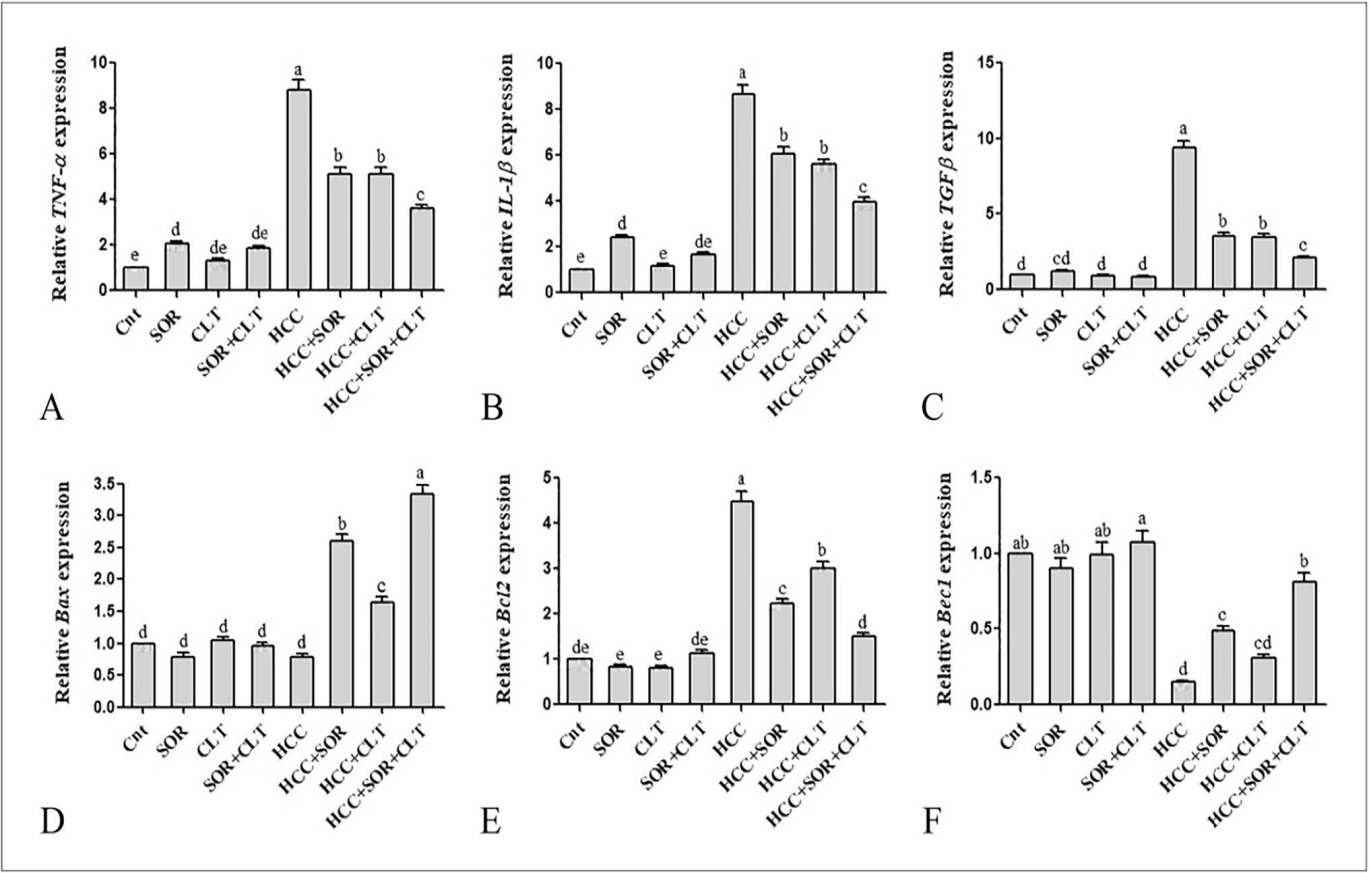
Gene expressions of **A** *TNF-α*, **B** *IL-1β*, **C** *TGF-β*, **D** *Bax*, **E** *Bcl2*, and **F** *Bec1* in liver tissues. The mRNA expression levels were measured by qRT-PCR and normalized to *β-actin*-binding protein. Data were expressed as mean ± SEM (*n* = 15). Small (a–e) letters depicted the statistically significant change at *P* ≤ 0.05 using ANOVA followed by post hoc Tukey test for multiple comparisons test. The significant changes are expressed by different letters above the columns. Abbreviation: SOR: sorafenib; CLT: dicinnamoyl-L- tartaric acid; HCC: hepatocellular carcinoma

CLT anticancer efficacy was confirmed by qRT-PCR analysis on the expression of inflammatory cytokines (*TNF-α*, *Il-1β*, *TGF-β*), apoptotic related gene (*Bax*), anti-apoptotic related gene (*Bcl2*), and autophagy-related-gene (*Bec1*). The qRT-PCR data revealed that the expression level of *TNF-α*, *Il-1β*, *TGF-β*, and *Bcl2* was significantly upregulated as well as significantly down-regulated of *Bax*, and *Bec1* in the HCC group in comparison with the untreated control group, while the administration of SOR and/or CLT significantly downregulated the expression level of *TNF-α*, *Il-1β*, *TGF-β*, and *Bcl2*, and significantly upregulated the expression of *Bax*, and *Bec1* compared to the untreated HCC group. The effect of a combination of SOR and/or CLT treatment is more effective than the effect of SOR alone or CLT alone treatment as revealed in Fig. 7.

## Discussion

The liver tumor in some patients does not respond to chemotherapy or radiotherapy, and difficult surgical removal of it may lead to patient death (Bruix and Sherman 2011). This is because these tumors have a high rate of metastasis, meaning they spread quickly to other parts of the body, making them difficult to treat with conventional methods. Additionally, the tumors can become resistant to treatment, making them even more difficult to remove. Recently, SOR, a potential drug approved by the FDA for treating advanced HCC, is an effective drug (Kim et al. 2017; Abdelgalil et al. 2019); however, it has resistance in some patients (Labeur et al. 2018). Thus, numerous natural substances and their secondary metabolites are being investigated to see if they have any anticancer or cancer-preventive properties (Yasin et al. 2015). This study is the first study that investigates the anticancer activity of a newly developed derivative of HCA named CLT in vivo and investigates if the co-treatment of CLT with SOR elevates the SOR’s efficacy or not. There was indicated that the hepatic tumor can be induced by the administration of the initiator (p-DAB), and the promoter (PB) for a long time (Aydinlik et al. 2001; Pathak and Khuda-Bukhsh 2007), while the administration of PB alone needs about 54–61 weeks to induce HCC (Akamatsu and Ikegami 1968). This previous study was consistent with our study, in which giving both DAB and PB to model mice for 2 months developed HCC. Liver damage has many indicators such as elevation of liver enzymes in the serum including ALT, AST, and GGT as well as other indicators of liver damage; Whitby et al. (1984) agree with our data, in which the administration of DAB/PB was induced elevation of liver function enzymes including AST, ALT, GGT, and ALP activity as well as elevation of serum AFP compared to normal control group either treated or untreated. Blood AFP level acts as a diagnostic tool for HCC (Sell 2008); thus, this confirms the hepatocellular carcinoma induction in mice mode in this study. The administration of CLT and/or SOR decreased the levels of AFP in our study. This is likely due to the fact that SOR and CLT have different mechanisms of action. SOR is known to inhibit cell proliferation and induce apoptosis of HCC cells (Huang et al. 2013), while CLT has been shown to modulate the expression of inflammatory mediators in the liver, which can reduce the levels of serum AFP (Ibrahim et al. 2020). Therefore, the combination of the two treatments is more effective in reducing inflammation than either of the treatments alone.

Many studies indicated that proinflammatory cytokines may be produced as a result of ROS (Angulo and Fresno 2002). Our data indicate that the administration of DAB/PB induced a significant elevation of the expression level of the inflammatory and antiapoptotic related genes including *TNF*-α, *IL-1β*, *TGF-β*, and *Bcl-2*, as well as significantly decrease the expression level of apoptotic and autophagy-related genes including *Bax* and *Bec1*. Our molecular data are consistent with another study that reported that in which exposure to DAB induces activation of nuclear factor (*NF*)-*kB*, which is a key regulator of inflammation and cell proliferation (Dolcet et al. 2005), involving the phosphorylation and degradation of the NF-кB inhibitor. Vascular endothelial growth factor-1 (*VEGF-1*), matrix metalloproteinase-9 (*MMP-9*), and *MMP-2*, as well as the expression of anti-apoptotic proteins like *Bcl-2* and *Bcl-XL*, are upregulated by *NF-kB*. On the other hand, pro-apoptotic proteins like *Bax*, *caspase-9*, and *caspase*-*3* are downregulated by *NF-kB* (Whitby et al. 1984; Murugan et al. 2010). Furthermore, the expression level of the *Beclin1* protein was downregulated in many types of cancers, and this suggests that there is a direct connection between Beclin1-induced autophagic cell death and tumor formation (Xia et al. 2013; Han et al. 2014), as well as *Beclin1* is an autophagy gene that can be downregulated to significantly inhibit autophagy, protecting tumor cells from autophagic cell death and promoting the growth of tumor cells (Wang et al. 2007).

HCC development was confirmed by histopathology analysis that showed atypical lesions of HCC; this may be due to the catabolism of DAB by cytochrome P-450 enzymes, which results in the creation of reactive oxygen species (ROS), toxic electrophiles, and DNA adducts that produce liver tumors (Thomas et al. 2016). Oxidative stress is a crucial factor in the growth of cancers (Kensler and Trush 1984), due to reactive oxygen and nitrogen species (ROS and RNS) that can interact with DNA bases to form promutagenic DNA adducts (Bartsch and Nair 2006). This study revealed that the administration of DAB/PB for 2 months significantly increased the level of the hepatic MDA and NO and significantly decrease the antioxidant activity of SOD and Gpx; this agrees with Rajkapoor and his colleagues (Rajkapoor et al. 2005) who reported that the lipid peroxidation (LPO) can produce many of harmful byproducts, including 4-hydroxy nonenal and MDA, which can assault DNA and cause mutagenicity and cancer. The previous reports documented that oxidative stress plays important role in liver damage and cancer (Kensler and Trush 1984).

In the same line, our histopathological data revealed that the liver tissue of mice that received DAB/PB has many pathological changes that indicate the HCC such as hepatocyte degeneration, loss of hepatic architecture, multiple lymphoid stroma aggregations, and congestion of hepatic blood vessels, and this indicates that exposure to DAB/PB can cause significant and extensive damage to the liver tissue, leading to the formation of HCC. This is further supported by the findings of Thomas et al. (2016), who identified additional changes in the liver, such as hyperplasia of the hepatic parenchyma cells, loss of contact inhibition, and damage of the central vein of the liver lobules.

The administration of any of a wide variety of chemical agents, both naturally occurring and synthetic, can induce phase 2 enzymes in numerous target tissues, which appears to be a sufficient requirement for attaining chemoprevention (Kensler 1997). Thus, antioxidants can bind to the free radicals produced by ROS and neutralize them, thus inhibiting the chain reaction of oxidative damage. Additionally, antioxidants can also signal to cells to increase the production of molecules that help protect them from oxidative damage (Firuzi et al. 2011). The previous study documented that HCAs and their derivatives have strong antioxidant activity due to the presence of a phenolic hydroxyl group, which can react with free radicals to form a resonance-stabilized phenoxyl radical and a propionic side chain, which has the potential to stabilize the phenoxyl radical via a conjugated double bond (Sova 2012; Kancheva 2009), and this finding agrees with our data, in which the administration of CLT and/or SOR induced a significant increase in the antioxidant activity of SOD and GP_X_ as well as significantly decrease the MDA and NO levels in comparison with the untreated HCC group. Furthermore, SOD is a mitochondrial enzyme that can neutralize free radicals and prevent tissue damage by the proportional splitting of two superoxide radicals in H_2_O_2_ and O_2_. Additionally, CAT also supports antioxidant enzymes by converting H_2_O_2_ to H_2_O (Attri et al. 2001).

Interestingly, our data showed that compared to the HCC group, the CLT treatment dramatically reduced the expression levels of *TNF-α*, *IL-1β*, *TGF-β*, and *Bcl2* and increased the expression of *Bax* and *Bec1*. This indicates that the CLT treatment was able to upregulate apoptotic pathways while downregulating inflammatory pathways, suggesting that it is a potential therapeutic option for certain types of cancer. Our results support prior research findings that HCA and its derivatives have anti-inflammatory properties (Alam et al. 2016; Da Cunha et al. 2004) and another recent study in rat chondrocytes documented that *p*-coumaric acid (HCA derivatives) inhibited *IL-1β*-induced inflammatory responses by blocking the *NF-kB* signaling pathways (Huang et al. 2020).

To conclude, our findings indicate that the CLT drug was able to modulate the inflammatory environment, activate autophagy-related pathways, and induce apoptosis, suggesting that this drug may be effective in treating diseases associated with inflammation. Furthermore, the CLT drug works by inhibiting the growth of new blood vessels that tumors need to grow and spread, whereas the SOR drug works by blocking certain proteins that tumors need to develop and proliferate. Both drugs were found to be effective in reducing tumor size and promoting tumor regression. Aside from that, CLT has been shown to increase the ability of SOR to penetrate cell membranes and enter the cells, allowing for more efficient delivery of the drug to the target cells. By co-delivering the two together, it is possible to more precisely target the cancer cells while reducing the drug’s exposure to healthy cells, leading to fewer side effects and improved therapeutic outcomes.

## Data Availability

Data are available upon request.
